# Electronic and magnetic properties of 2D vanadium-based transition metal dichalcogenides

**DOI:** 10.1038/s41598-023-48141-1

**Published:** 2023-11-28

**Authors:** Mirali Jafari, Wojciech Rudziński, Józef Barnaś, Anna Dyrdał

**Affiliations:** 1 Department of Mesoscopic Physics, ISQI, Faculty of Physics, Adam Mickiewicz University in Poznań, ul. Uniwersytetu Poznańskiego 2, 61-614 Poznań, Poland; 2 Institute of Molecular Physics, Polish Academy of Sciences, ul. M. Smoluchowskiego 17, 60-179 Poznań, Poland

**Keywords:** Condensed-matter physics, Two-dimensional materials

## Abstract

In this paper, electronic and magnetic properties of monolayers and bilayers of Vanadium-based transition metal dichalcogenides VX_2_ (X = S, Se, Te) in the H phase are investigated theoretically using methods based on DFT calculations as well as analytical methods based on effective spin Hamiltonians. The band structure has been computed for all systems, and then the results have been used to determine exchange parameters and magnetic anisotropy constants. These parameters are subsequently used for the determination of the Curie temperatures, hysteresis curves, and energy of spin-wave excitations. In the latter case, we compare analytical results based on effective spin Hamiltonian with those determined numerically by Quantum ATK software and find a good agreement. The determined Curie temperature for VTe_2_ monolayers and bilayers is below the room temperature (especially that for bilayers), while for the other two materials, i.e. for VS_2_ and VSe_2_, it is above the room temperature, in agreement with available experimental data.

## Introduction

Van-der-Waals (vdW) magnetic materials are currently of great interest as they are considered as future materials for building two-dimensional (2D) electronic and spintronic devices, including atomically thin spin valves, nonvolatile memory elements, and gates for information processing. The most fascinating aspects of the physics observed in van-der-Waals materials are the magnetism and magnetic phase transitions emerging in monolayers and in a few layers of these 2D crystals. The discovery of ferromagnetism in single layers of CrI_3_^1^ and Cr_2_Ge_2_Te_6_^2^ initiated an enormous interest in magnetic 2D vdW crystals. These crystals now serve as a large platform for basic research on 2D magnetism, and additionally allow to mimic the physics of ideal Ising, Kitaev or Heisenberg models and magnetic phase transitions induced by external gate voltages or strain. The magnetic ground state of 2D vdW crystals depends, among others, on their crystallographic phase, stacking geometry, and possible twisting of adjacent monolayers. The current worldwide interest in magnetic vdW crystals follows not only from their novel and interesting physics, but also from their potential for applications (e.g. in quantum computing, topological magnonics, spintronics, optoelectronics, and others)^3^.

**Figure 1:**
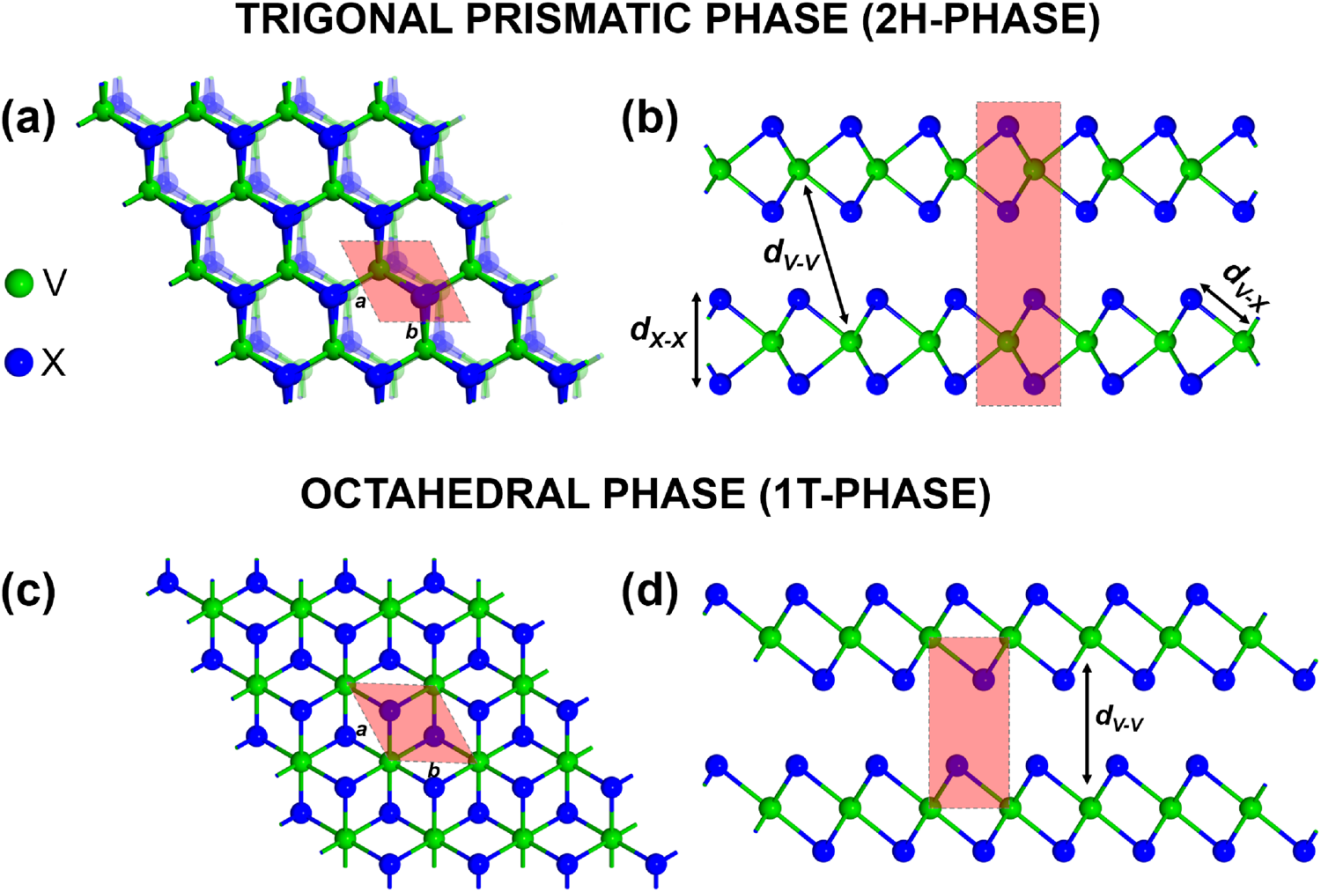
Schematic structure of the 2H phase of VX_2_ monolayer and bilayer structures. Top (**a**) and side (**b**) views, as well as the elementary units are shown. Various bondlengths are also indicated in (**b**). For comparison, the top and side views of the T-phase bilayer are also shown in (**c**) and (**d**).

Recently, two main groups of magnetic vdW materials have been focusing great attention: transition metal trihalides, with CrI_3_ being thought of as a prototype of 2D magnetic crystal, and magnetic transition metal dichalcogenides (TMDCs). In this paper, we focus on Vanadium-based dichalcogenides, VX_2_ (X = S, Se, and Te). Two different polymorphs of VX_2_ materials are currently known; the trigonal prismatic crystallographic structure (2H, D3h) and the octahedral (1T, D3d) one^4–6^, as presented schematically in Fig. 1. Additionally, a distorted octahedral (1T$_d$) phase has been identified^7^. These materials consist of monolayers of VX_2_, that are weakly coupled by van der Waals forces and therefore can be easily exfoliated down to a single monolayer or a few-layer form. A monolayer of VX_2_ material consists of a hexagonal atomic plane of Vanadium atoms, that is sandwiched between two chalcogen (X) atomic planes. However, the positions of X atoms are different in different phases. The corresponding unit cell includes one Vanadium atom and two chalcogen atoms. In the case of bilayers (BLs) of the hexagonal 2H phase, the V (X) atoms of the top layer are above the X (V) atoms of the bottom layer. In turn, in the bilayers of the T1 phase, the V atoms of the top layer are above the V atoms of the bottom layer. This can be clearly seen in the top and side views of the bilayers, shown in Fig. 1.

While there are several theoretical studies on the monolayers and bilayers of VX_2_ materials in the 2H phase^8,9^, and these materials were successfully synthesized in the bulk and multilayer forms^6^, VSe_2_ and VTe_2_ in the trigonal (1T) phase have been synthesized only in the monolayer form.^10–13^. Therefore, in this paper, we present the results of our detailed analysis of the monolayers and bilayers of Vanadium-based transition metal dichalcogenides, VX_2_ (where X = S, Se, Te), in the H-phase only. In the beginning, we focus on electronic properties, especially on the band structures of these materials. Then, we evaluate the basic magnetic parameters, like magnetic moments of Vanadium atoms, exchange parameters, and magnetic anisotropy constants. These parameters are subsequently used in the simulations of Curie temperature, magnetic hysteresis curves, and spin wave excitations. Details of the methods used in calculations are described in section METHOD.

We also note that though there is relatively broad literature on TMDCs, their behavior, especially in the monolayer and bilayer forms, is still under discussion. This concerns even the magnetic ground state as numerical results based on DFT calculations depend on many approximations and assumptions. Our results present a contribution to this discussion—especially our investigation of bilayers with antiferromagnetic coupling between the two monolayers is of particular interest, as such bilayers are natural candidates for 2D spin valves.

## Results

### Atomic structure

Figure 1a and b show respectively the top and side views of the atomic structure in a bilayer of H-phase dichalcogenides VX_2_. Each Vanadium atom is surrounded by six nearest-neighbour chalcogen atoms, and the corresponding lattice parameters *a* and *b* (see Fig.1a) are equal, $a=b$, in honeycomb structures. The structural lattice parameters and bondlengths after optimization are presented in Table 1 for both monolayers and bilayers.

**Table 1:** Optimized lattice constants *a*, and the bondlengths $d_{V-X}$, $d_{V-V}$, and $d_{X-X}$ between various atoms, as defined in Fig. 1.

VX_2_	N	*a* [Å]	${d_{{V-V}}}$ [Å]	${d_{{V-X}}}$ [Å]	${d_{{X-X}}}$ [Å]
VS_2_	1	3.17159	-	2.36	2.98
	2	3.17147	6.30	2.36	2.97
VSe_2_	1	3.31934	-	2.49	3.20
	2	3.31971	6.66	2.49	3.19
VTe_2_	1	3.59006	-	2.72	3.53
	2	3.59069	7.15	2.71	3.52

N indicates the number of layers, N = 1 for monolayers and N = 2 for bilayers.

From this table follows that there is no significant change in the lattice parameters when the number of layers increases from N = 1 to N = 2. However, when the atomic number of chalcogen atoms increases, the lattice constant increases as well. Additionally, the bondlengths between Vanadium and X atoms ($d_{V-X}$) and between the chalcogenide atoms ($d_{X-X}$) also increase with the increasing atomic number of chalcogen atoms. The obtained results are in very good agreement with earlier studies^8,14^. From Table 1 also follows, that the bondlengths are rather weakly modified when the number of layers N increases from 1 to 2, similarly as in the case of lattice parameters, where the change is only 0.31%. However, the changes associated with different X atoms can be up to 12.91% and are quite significant. This is a consequence of the increasing radius of atomic orbitals with increasing atomic number, which in turn leads to increasing bondlengths and subsequently weaker bonding in VTe_2_ as compared to VS_2_. The intralayer distance between two nearest-Vanadium atoms is remarkably different from the interlayer one. The optimized structures, including also the Van-der-Waals correction, show that the largest interlayer distance is for VTe_2_ while the lowest distance is for VS_2_.

### Electronic bandstructure

**Figure 2:**
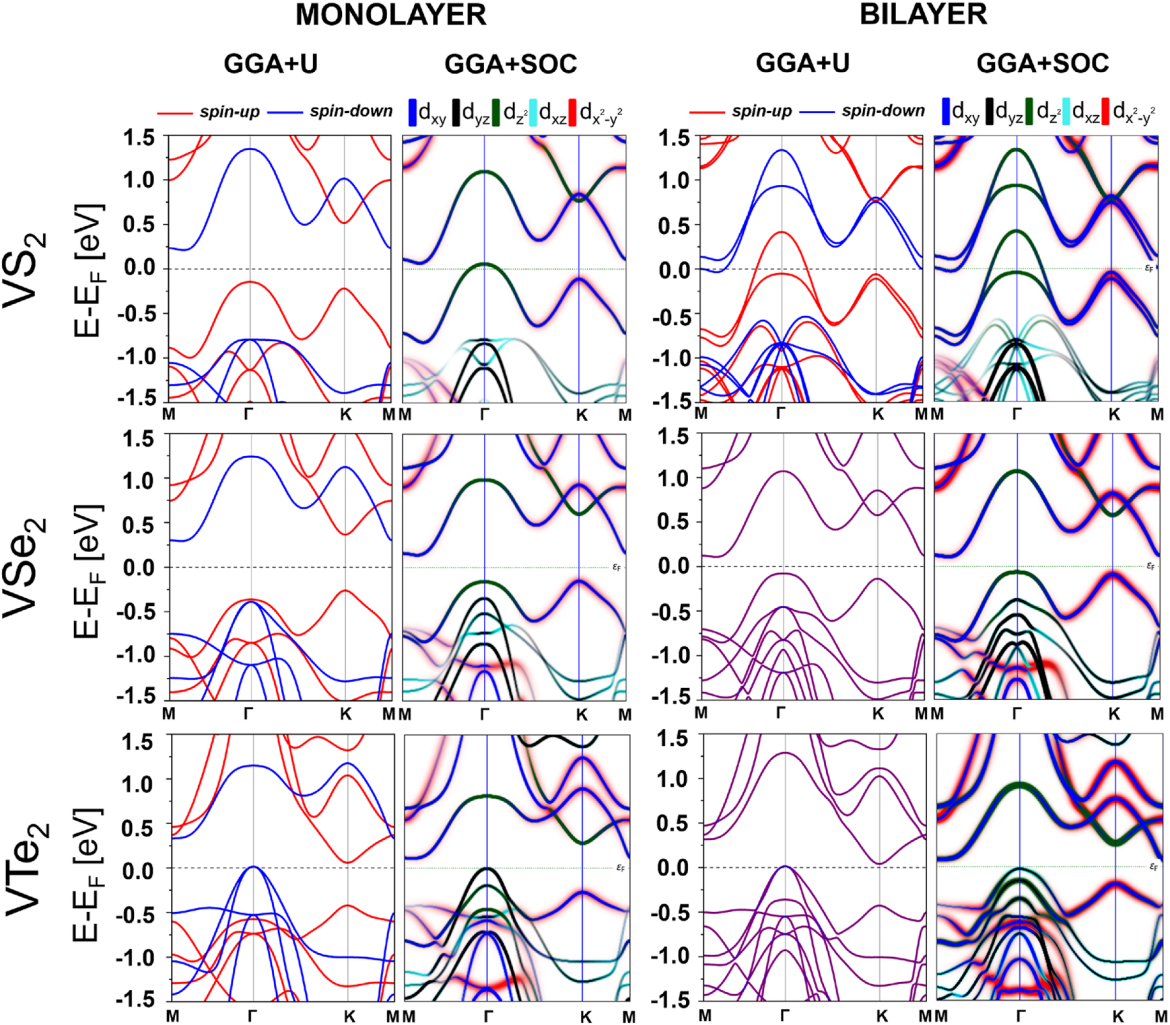
Spin-resolved band structures for the monolayers (first and second columns from left) and bilayers (third and fourth columns from left) of VX$_2$ (X = S, Se, Te) in the GGA+U approximation (first and third columns) and GGA + SOC approximation (second and fourth columns) where $d-$orbital-decomposition is added to visualize contributions of the Vanadium orbitals near the Fermi energy $E_{F}$.

Before calculating spin-resolved electronic band structures of the VX$_{2}$ (X = S, Se, and Te) monolayers and bilayers, one needs to determine first the corresponding magnetic ground states. Therefore, for each system we calculated its total energy in three different spin configurations: non-magnetic (NM), ferromagnetic (FM), and antiferromagnetic (AFM). In all calculations, the NM state was the most unstable one. Then, we calculated the total energy difference between the FM and AFM states, $\Delta E=E_{AFM}-E_{FM}$. The negative sign of $\Delta E$ indicates that the AFM state is stable, while the positive sign shows the FM state is stable. The obtained ground states for all systems under consideration are given in Table 2. From these data follows that all the monolayers have a ferromagnetic ground state. However, for the bilayer structures, different materials may have different ground states. The total energy in the antiferromagnetic spin configuration of VS_2_ is larger than that in the ferromagnetic case, so the ground state is FM. On the contrary, for VSe_2_ and VTe_2_, we found that magnetic moments of the two V atomic planes are arranged antiferromagnetically in the ground state, i.e. the interlayer exchange coupling is antiferromagnetic, in agreement with other works^9,15–18^.

**Table 2:** Calculated ground states, magnetic moments (absolute values) of V atoms, and energy band gaps.

$VX_2$	N	Ground state	Magnetic moment of V [$\mu _{B}$]	Bandgap [eV]	
			GGA + U	GGA + U	GGA + SOC
$VS_2$	1	FM	1.267	0.35	Metallic
	2	FM	1.228	Half-metal	Metallic
$VSe_2$	1	FM	1.435	0.55	0.29
	2	AFM	1.437	0.50	0.17
$VTe_2$	1	FM	1.679	Half-metal	0.11
	2	AFM	1.709	Metallic	0.10

N indicates the number of layers.

From the DFT calculations, we have determined the magnetic moments of Vanadium atoms for all the systems under consideration. These moments have been calculated within the GGA approximation with Coulomb correction U included, referred to in the following as the GGA + U approximation. The Hubbard parameter U describes the on-site Coulomb interaction between electrons, and the impact of finite U on electronic spectrum in VX_2_ dichalcogenides was already studied in the relevant literature. Especially, Ref. ^19^ gives clear arguments that U = 2 is an appropriate approximation for VX_2_ materials. Following this paper, as well as other relevant pulications ^8,20–22^, we assumed U = 2 eV in our numerical calcuations. The obtained magnetic moments of Vanadium are presented in Table 2. From this table and also from other calculations (not shown here) follows that the Vanadium ion preserves absolute value of its magnetic moment when the number of layers varies from one to two—independently of the approximation used in calculations. However, the Coulomb correlation U leads to a slightly larger magnetic moment in comparison to that obtained in the GGA calculations with and without spin-orbit coupling (SOC) included. From our calculations follows that the magnetic moment of V ion in monolayers is the lowest one for VS_2_, where is equal to 1.267 $\mu _B$ ($\mu _B$ is the Bohr magneton). In turn, in VSe_2_ and VTe_2_ it is 1.435 $\mu _B$ and 1.679 $\mu _B$, respectively.

Figure 2 shows the electronic band structures of the monolayers and bilayers of VX_2_ along the high symmetry points in the Brillouin zone. For both, monolayers and bilayers, the calculations are based on the GGA + U approximation, and additionally we also show there the band structures in the GGA approximation with spin-orbit interaction included (GGA + SOC approximation). From comparison of the results for GGA (not shown) and GGA + SOC approximations follows that the spin-orbit interaction does not lead to significant modifications in the band structure. This is also consistent with the results obtained in Ref. ^8^.

In the case of band structures calculated in the GGA+SOC approximation, we distinguish in Fig. 2 contributions from the V and X atoms. Generally, these results demonstrate that the main contribution to the bands near the Fermi level comes from the Vanadium atoms. As illustrated in Fig. 2, in the VS_2_ monolayers and bilayers, the orbital $d_{z^{2}}$ of Vanadium has a large contribution to the valance band maximum (VBM) near the $\Gamma$ point, while for the conduction band minimum (CBM) the Vanadium $d_{xy}$ orbital dominates at the *M* point. A similar situation happens also in the monolayers and bilayers of VSe_2_, but with the contribution of $d_{x^{2}-y^{2}}$ orbitals of Vanadium dominating for the VBM at the *K* point and a combination of the $d_{z^{2}}$ and $d_{xy}$ orbitals dominating for the CBM at the *M* point. Regarding the VTe_2_, the mixture of $d_{yz}$ and $d_{xz}$ states of Vanadium dominates for the VBM at the $\Gamma$ point, while the $d_{z^{2}}$ and $d_{xy}$ orbitals dominate for the CBM around the *M* point.

Basic features of the electronic structure obtained in the GGA+U and GGA+SOC approximations, especially the bandgaps, are listed in Table 2. From this table follows that in the GGA+U approximation, the ferromagnetic VS_2_ monolayers display semiconducting behaviour (with the gap of 0.35 eV), while the bilayers are then half-metallic due to a finite density of states at the Fermi level in the spin-up band. In turn, in the GGA + SOC calculations, both monolayers and bilayers have metallic properties. In the case of VSe_2_, both monolayers and bilayers reveal semiconducting behaviour with the gaps in the GGA + U calculations equal to 0.55 eV for the monolayers and 0.50 eV for the bilayers. This behaviour also appears in the GGA + SOC calculations, but with the band gaps reduced to 0.29 eV and 0.17 eV for the monolayers and bilayers, respectively. However, the monolayers of VTe_2_ display half-metallic behaviour in the GGA + U calculations, while the bilayers are then metallic. In the GGA + SOC calculations, both monolayers and bilayers are semiconducting with narrow gaps equal to 0.11 eV for the monolayer and 0.10 eV for the bilayer structures.

When comparing results of various approximations, we arrived at the conclusion that including spin-orbit coupling (SOC) does not make significant quantitative changes in the band structure, though some qualitative differences may appear due to spin mixing induced by SOC. We verified that GGA + SOC calculations give results which are quantitatively and also qualitatively similar to those obtained in the GGA approximation (not shown). From Ref. ^8^ follows that the results of GGA+U+SOC calculations are quantitatively similar to those of GGA + U. In turn, including Coulomb correlation U into calculations may lead to remarkable changes in the electronic spectrum, especially in the band gaps. From the above observations one may conclude that when calculating properties that do not originate from SOC, the approximation GGA + U seems to be the appropriate one. In turn, when a particular property (like for instance. magnetic anisotropy) originates from SOC, then in the first approximation GGA + SOC is sufficient.

### Magnetic anisotropy energy

**Figure 3:**
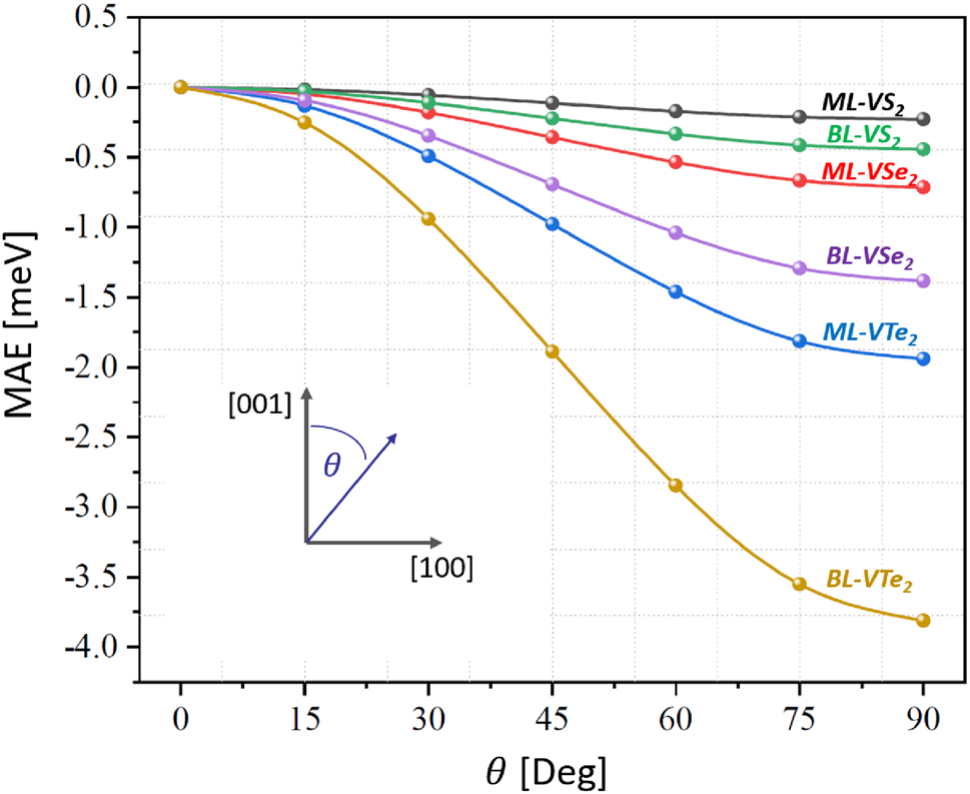
Angular dependence of the magnetic anisotropy energy per unit cell, MAE, of monolayers (ML) and bilayers (BL) of V-based TMDs. The anisotropy energy is shown as a function of the polar angle $\theta$ for the azimuthal angle equal to 0 (see the inset for the definition of $\theta$).

Magnetic anisotropy energy (MAE)  is an important parameter that determines the magnetisation orientation in the ground-state. This anisotropy originates from the spin-orbit coupling and can be calculated from the energy difference between relevant spin orientations using the force theorem. This theorem allows one to evaluate MAE using non-self-consistent band energies, and in general, it is defined as follows^23–25^:

1 $$\begin{aligned} MAE = \sum _i f_i(\theta _1, \phi _1) \epsilon _i(\theta _1, \phi _1) - \sum _i f_i(\theta _0, \phi _0) \epsilon _i(\theta _0, \phi _0), \end{aligned}$$

where $f_i(\theta , \phi )$ refers to the occupation factor for the band *i* (*i* includes also k-point index), with $(\theta , \phi )$ and $\epsilon _i(\theta , \phi )$ describing the spin orientation and the corresponding band energy, respectively. In this paper, we focus on the perpendicular (out-of-plane) and in-plane anisotropies. When the system is magnetically isotropic (or nearly isotropic) in the plane, then the perpendicular anisotropy is defined as $MAE=E_{[100]}-E_{[001]}$ (or $MAE=E_{[010]}-E_{[001]}$), with the *x*, *y* axes in the layer plane and the *z* axis perpendicular to the layer. Thus, positive value of *MAE* corresponds to the perpendicular easy axis, while negative value to the perpendicular hard axis, i.e. to easy-plane anisotropy.

Table 3 shows MAE (per unit cell) for the materials considered in this paper. The perpendicular MAE for the monolayer of VS_2_, VSe_2_, and VTe_2_ is − 0.2276 meV, − 0.7143 meV, and − 1.9431 meV, respectively. The MAE of the bilayers, is approximately twice as large as that of the corresponding monolayer. The calculations show, that MAE has a negative sign for the monolayers and bilayers, which indicates that the anisotropy is of easy-plane type. The dependence of MAE on the polar angle (angle between magnetization orientation and axis perpendicular to the layer plane) is shown in Fig. 3 for all the systems under consideration.

As presented in Table 3, we have also determined the in-plane easy-axis MAE which is defined as the difference between two in-plane magnetization directions namely, $MAE=E_{[100]}-E_{[010]}$. Because of the symmetry of the studied systems, this anisotropy is negligibly small, of an order of micro/nano eV, which indicates its insignificant role in the magnetization processes, though it determines the magnetization orientation in the zero temperature limit. We note, that our calculations are for unstrained structures. It is however known that external strain has a significant impact on the magnetic anisotropy, and can remarkably change the anisotropy constants.

### Exchange parameters, Curie temperature and hysteresis loops

**Table 3:** Calculated perpendicular and in-plane magnetic anisotropy energy (MAE) per unit cell within the GGA + SOC approximation, and the exchange coupling parameters $J_1, J_2, J_{int}$ within the GGA + U approximation.

$VX_2$	N	*MAE*		$J_{ij}$ [meV]		
		Perpendicular [meV]	In-plane [neV]	$J_1$	$J_2$	$J_{int}$
$VS_2$	1	− 0.22	3.58	25.68	− 2.13	–
	2	− 0.44	− 0.12	26.67	− 1.63	0.50
$VSe_2$	1	− 0.71	− 15.28	19.52	− 0.94	–
	2	− 1.38	− 10.69	18.60	− 1.15	− 0.05
$VTe_2$	1	− 1.93	− 35.79	9.84	− 0.78	–
	2	− 3.81	18.93	8.69	− 0.66	− 0.11

A very important parameter, especially when one considers application possibilities, is the Curie temperature $T_C$. In this paper, we evaluate $T_C$ of VX$_{2}$ monolayers and bilayers within two different methods: the mean-field approximation (MFA)^26^ and the Monte–Carlo (MC) simulations. The corresponding calculations are based on the Heisenberg spin Hamiltonian with classical spins:

2 $$\begin{aligned} H = -\sum _{i,j}J_{ij}S_i.S_j -K\sum _{i}{(S_{i}^{z})^{2}}, \end{aligned}$$

where $J_{ij}$ is the exchange coupling constant between V atoms located in the sites *i* and *j*, $S_i$ is the local spin vector on the atom *i*, while *K* is the easy-plane magnetic anisotropy constant, $K<0$. The anisotropy constant *K* is determined by the above calculated *MAE*. We take into account only the exchange coupling $J_{ij}$ between nearest neighbours, $J_1$, and next-nearest-neighbours, $J_2$, for the intralayer interactions. In the case of bilayer structures, we also include interlayer coupling $J_{int}$. All the exchange parameters have been determined using the energy mapping method^27^. Contrary to the other methods, where results rely on the total energy analysis, in this method one calculates the exchange constant for a particular wave vector *q* using the magnetic force theorem (for more details see Ref. ^26^). Table 3 shows all the exchange constants for the monolayers and bilayers of the materials under consideration, obtained in the GGA + U approximation. From this table follows that the largest exchange parameters are for VS_2_, and the smallest ones for VTe_2_. For the bilayers we have also determined the exchange parameter $J_{int}$ between the two monolayers and found $J_{int} =$0.5 meV, -0.05 meV, and -0.11 meV for VS_2_, VSe_2_, and VTe_2_, respectively. Positive and negative signs of $J_{int}$ correspond to the FM and AFM interlayer coupling, respectively.

The Curie temperature, $T_C$, of a ferromagnet is a temperature at which the average magnetic moment becomes equal to zero due to random thermal fluctuations of the local magnetic moment orientations. With the obtained exchange parameters, the Curie temperature could be estimated using the mean field approximation method (MFA) as follows:^26^

3 $$\begin{aligned} T_{C}^{\text {MFA}} = \frac{2}{3k_B}\sum _{j{\textbf {R}} \ne i{\textbf {0}}} J_{i{\textbf {0}},j{\textbf {R}}}, \end{aligned}$$

where $J_{i{\textbf {0}}j{\textbf {R}}}$ stands for the exchange coupling parameter between the atom at site *i* in the main considered primitive cell (labeled with 0) and the atom at site *j* in a different unit cell displaced from the central one by the lattice vector ${{\textbf {R}}}$. To find the Curie temperatures in the MFA, listed in Table 4, we performed GGA+U calculations up to the third nearest neighbours. Interestingly, the results reveal Curie temperatures close to or beyond the room temperature for most of the analyzed structures. In general, the mean-field approximation overestimates $T_C$ when compared to experiment and other methods. This appears because the mean-field approximation neglects the role of magnetic fluctuations in the system, which in 2D are relatively large. However, $T_C^{MFA}$ can be considered as the upper limit of Curie temperature, and we show it in Table 4 for comparison.

**Table 4:** Curie temperature $T_C$ obtained within the mean field approximation (MFA) and Monte–Carlo (MC) simulations, as well as the corresponding power factor $\beta$.

$VX_2$	N	$T_{C}$ [*K*]		
		MFA	MC	$\beta$
$VS_2$	1	438.72	289.45	0.48
	2	446.95	333.06	0.39
$VSe_2$	1	434.01	249.69	0.46
	2	416.97	229.10	0.41
$VTe_2$	1	296.39	126.61	0.38
	2	268.44	119.57	0.39

**Figure 4:**
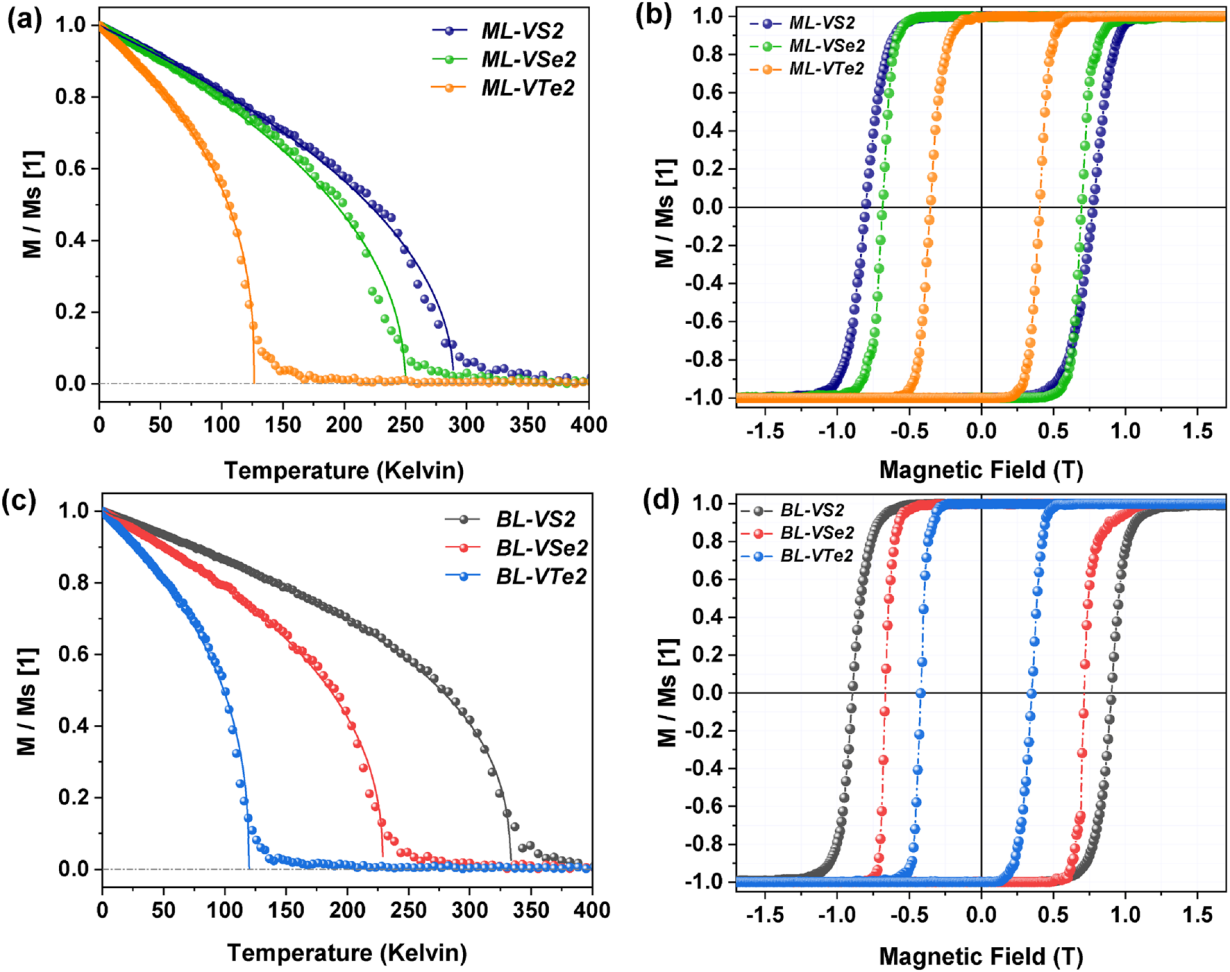
Magnetization normalized to the saturation magnetization $M_s$ as a function of temperature within the Monte Carlo simulations for monolayers (**a**) and bilayers (**c**) of $VX_2$, and the corresponding hysteresis curves (**b**, **d**).

To get more realistic values of $T_C$, we used the atomistic Vampire code package^28, 29^ as well as the magnetic moments, MAE, exchange parameters $J_1$, $J_2$, and $J_{int}$ obtained from the DFT calculations, and simulated magnetization vs. temperature for VX$_2$ monolayers and bilayers within the Monte Carlo metropolis algorithm^30,31^. The results are shown in Fig.4. To fit the magnetisation versus temperature curve, Fig. 4a and c, the Curie–Bloch equation in the classical limit was used:

4 $$\begin{aligned} M(T)=M_s\biggl ( 1-\frac{T}{T_{C}}\biggr )^{\beta }, \end{aligned}$$

where *T* is the temperature and $T_{C}$ is the Curie temperature, while $\beta$ is the critical exponent, which is generally different for different materials. As follows from Table 4, the Curie temperature determined by the Monte–Carlo calculations is lower than that in the mean field approximation. Anyway, all the results show that the highest $T_C$ is for VS$_2$ and the lowest one is for VTe$_2$ materials. This difference originates from the corresponding difference in exchange coupling parameters $J_1$ and $J_2$.

Other important characteristics of magnetic materials are the relevant hysteresis loops. These loops reveal magnetization reversal processes in an external magnetic field and therefore contain information on the magnetic ground state and possible magnetic phase transitions induced by the magnetic field. In Fig. 4b and d we present the hysteresis curves for the monolayers and bilayers of VX_2_.

In the simulation procedure, the system is slowly cooled down until the temperature reaches the desired value in the presence of a magnetic field *H* = 1T applied in the plane of the system and along the in-plane easy-axis (corresponding to a very small anisotropy). Then, the obtained spin configuration in the last step of cooling is used as the initial configuration for calculating the hysteresis curve.

Because individual monolayers are ferromagnetic, shapes of the hysteresis loops of all monolayers in Fig. 4 are similar and typical for ferromagnetic layers. As follows from Fig. 4, hysteresis loops for bilayers are similar to those for the corresponding monolayers, which indicates that the antiferromagnetic interlayer coupling does not play an important role in the hysteresis curves. This is because interlayer coupling is relatively small. For VTe_2_, e.g, the energy of interlayer coupling corresponds roughly to 10 K and is much smaller than the temperature for which the hysteresis curves were calculated (50 K) and also significantly smaller than the corresponding anisotropy energy (around 38 K). An interesting feature of the hysteresis loops is the coercivity, which is the largest for VS_2_ and smallest for VTe_2_, just opposite to the behavior of easy-plane anisotropy constant, which is the largest for VTe_2_ and smallest for VS_2_. The stronger anisotropy, the more fixed is the magnetic moment to the layer plane. Accordingly, magnetization rotation is in the layer plane, where in-plane anisotropy is very small.

### Dynamic properties: magnon spectra

**Figure 5:**
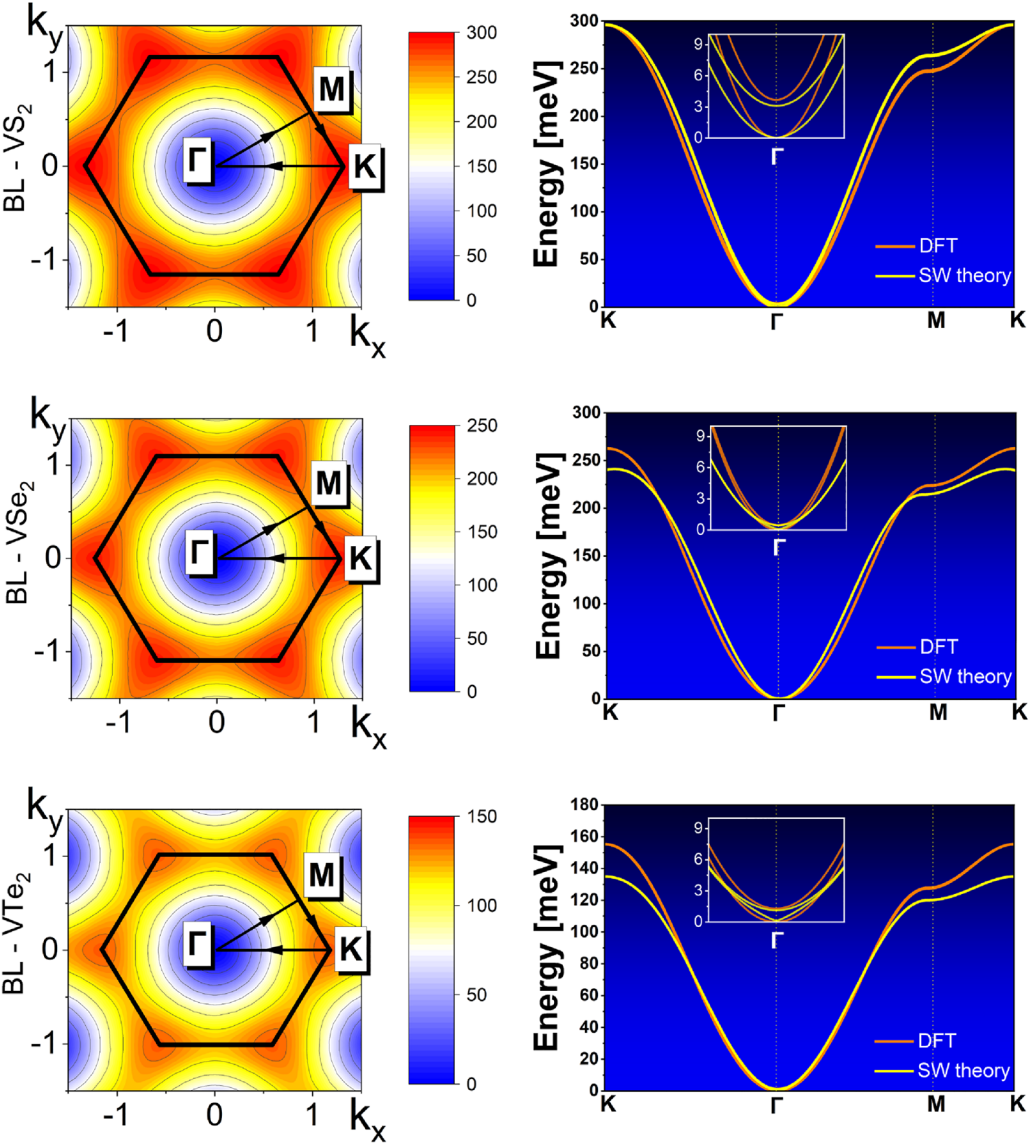
(Right panel) Magnon spectra in the VX_2_ bilayers (X = S, Se, Te). The red lines correspond to the numerically calculated spectra using the ATK package, while the yellow lines present the spectra obtained from the analytical model. The insets show the spectra in the vicinity of the Brillouin zone center ($\Gamma$ point), where splitting of the modes can be clearly seen. (Left panel) Density plots representing analytically calculated energy of spin waves in the whole Brillouin zone.

Having found the exchange and anisotropy parameters, determined from the calculated band structures, one can calculate the energy (frequency) of the collective excited states of the magnetic moments (spins), known as spin waves or magnons. We have determined the spectra of spin waves using the corresponding code implemented into the Quantum ATK package. Dispersion curves of these modes in bilayers along basic orientations in the Brillouin zone are shown in Fig. 5 (right column), where these spectra are compared with the corresponding spectra obtained analytically, with the parameters taken from DFT calculations. The energy of spin waves determined from the analytical model in the whole Brillouin zone is presented in the left column of Fig. 5 for all the three systems considered in this paper.

The analytical calculations are based on the effective spin Hamiltonian (2), generalized by including the in-plane anisotropy:

5 $$\begin{aligned} H = -\sum _{i,j}J_{ij}S_i\cdot S_j -K\sum _{i}{(S_{i}^{z})^{2}} -K_y\sum _{i}{(S_{i}^{y})^{2}}. \end{aligned}$$

Here, apart from the easy-plane anisotropy ($K<0$), there is also a weak in-plane easy-axis anisotropy along the axis *y*, with $K_y$ being the relevant anisotropy constant, $K_y>0$. This anisotropy constant is very small in the systems under consideration (see Table 3) and does not lead to significant features in the spectra. Additionally, in the analytical model the exchange interaction is limited to the intralayer and interlayer nearest-neighbours. The intralayer (ferromagnetic) and interlayer (antiferromagnetic) exchange parameters between Vanadium atoms are taken from DFT calculations and are adapted to the spin 1/2 model (spin of Vanadium atoms). Due to the easy-plane anisotropy, the magnetic moments of vanadium atoms in equilibrium are oriented within the atomic planes. The magnon modes are determined by assuming small deviations of the spin moments from their ground state orientation. Then, the spin operators are transformed into the local magnon operators using Hollstein-Primakoff transformation. The local-magnon Hamiltonian is finally diagonalized by the Fourier transformation followed by the Bogolibov transformation. More details on this procedure will be presented elsewhere^32^.

Figure 5 shows the energy of spin waves in all the bilayer systems studied in this paper. Each monolayer separately supports one magnon branch, as the monolayer contains only one magnetic atom in the elementary unit cell. Therefore, in the case of bilayers, there are two spin-wave modes, which slightly differ in energy due to coupling between the monolayers. In the absence of magnetic field and in-plane anisotropy (which is extremely small in the systems under consideration), the energy of the lower branch vanishes at the $\Gamma$ point ($\varvec{k}=0$) for all three systems, while the energy of the second mode in the $\Gamma$ point is nonzero, and the gap between these two modes is generally determined by the interlayer exchange coupling and easy-plane magnetic anisotropy. This gap is very small and can not be distinguished in the main parts of the spectra in Fig. 5, but it is well resolved in the insets presenting the zoomed-out areas around the $\Gamma$ points. This gap is clearly visible there for both analytical and computational spectra.

From Fig. 5 follows that the agreement between the modes calculated by the ATK package and by the analytical model based on some effective spin Hamiltonian is quite satisfactory. The largest deviations occur for VTe_2_, and especially at the Brillouin zone boundaries. It is obvious that the spectra obtained by these two approaches may differ due to some differences in the models. For instance, the ATK code includes the influence of further neighbours which is not taken into account in the analytical model. Despite this, the agreement is satisfactory. In addition, a comparison of the spectra obtained numerically with the analytical ones allows to prove the proper behaviour of the numerical spectra near the $\Gamma$ point of the Brillouin zone.

## Discussion

The van der Waals magnetic materials are expected to have a big impact on the development of future nanoelectronics and nanospintronics. These materials can be easily achieved in atomically thin layers, which makes them ideal building elements for novel two-dimensional electronics/spintronic devices. The studied monolayers and bilayers of Vanadium-based dichalcogenides, especially of VS_2_ and VSe_2_, are magnetic also at room temperatures and moderately above. Therefore, they can be considered as being prospective ones for certain applications. However, for real practical applications, the Curie temperatures should be still higher.

The antiferromagnetically exchange-coupled bilayers of the studied materials can be considered as natural spin-valves, in which the two monolayers have opposite magnetic moments at zero field. External field can rotate these moments from antiparallel to parallel configuration and this may be associated with a resistance change. Alternatively, one can build spin-valves by connecting two stripes of the two-dimensional ferromagnetic material with a stripe of two-dimensional nonmagnetic material. In fact, there are various possible architectures of the spin valves based on van der Waals materials. Unfortunately, the interlayer exchange coupling in vanadium-based TMDs is rather small, of the order of 1 meV. Accordingly, spin valves based on these materials can work at low temperatures, of the order of 10 K. For most of practical applications, however, one needs spin valves working at room temperatures. Therefore, further research on the already known materials, as well as a search for novel materials with better characteristics, is still required.

## Method

### DFT calculations

All the first-principles calculations in this paper have been performed in the framework of Density Functional Theory (DFT) using the *Quantum ATK* code package (version 2021.06-SP2)^33^, which is based on Hohenberg-Kohn theorem^34^ as well as the Kohn–Sham equations^35^. To expand the wave function, the SG15 collection of optimized norm-conserving Vanderbilt (ONCV) pseudopotentials with the Ultra Linear Combination of Atomic Orbitals (LCAO-U)^36^ basis set has been employed. We used the generalized-gradient approximation (GGA) in the formalism of Perdew–Burke–Ernzerhof (PBE) for the exchange-correlation interaction of electrons^37^. The energy mesh-cutoff of 600 eV within the total energy convergence criteria of $1 \times 10^{-6}$ eV ($10^{-8}$ eV for the magnetic anisotropy energy calculations) for each primitive cell has been performed. The two-dimensional Brillouin zone was sampled by a $\Gamma$-centered Monkhorst–Pack method^38^ using the k-point grid of 30 × 30 × 1. All studied structures have been fully optimized and minimized until the force on each atom is smaller than 0.02 eV/Å. To avoid any artificial interaction between image layers along the non-periodic directions, we employed at least 25 and 30 Angstroms vacuum layers for monolayer and bilayer structures, respectively. Moreover, to optimize the lattice parameters as well as the bondlengths, we have included a weak and non-local van der Waals (vdW) interaction between the layers in bilayer structures of VX_2_ materials. Using the so-called dispersion interactions, inter-layer bondlengths are decreased and the layers are not able to bind strongly and are separated. In our calculations, we used the semi-empirical corrections by Grimme DFT-D2^39^. To the electron-electron correlation effect of the localized 3*d* orbitals of Vanadium (V), we implemented the DFT+U calculations, in which the *U* term refers to the effective potential of the onsite Coulomb interaction for the V-3d electrons^40^.

### Static magnetic properties

To obtain magnetization as a function of temperature, and estimate Curie Temperature we have used the *Vampire* code based on the Monte Carlo metropolis algorithm (MC). For the MC calculations, we selected a $l\times l$ where *l* = 120 is the number of repeated units of the supercell with the periodic boundary conditions in all directions. We also used 10000 equilibrium steps followed by 10000 averaging steps.

## Data Availability

All relevant data are included in the article.

## Code availability

The calculations were performed using the Quantum ATK code package (version 2021.06-SP2), Vampire packages, and Wolfram Mathematica.
